# Between Technological Utopia and Dystopia: Online Expression of Compulsory Use of Surveillance Technology

**DOI:** 10.1007/s11948-024-00483-3

**Published:** 2024-05-15

**Authors:** Yu-Leung Ng, Zhihuai Lin

**Affiliations:** 1 Department of Interactive Media, Hong Kong Baptist University, Kowloon, Hong Kong SAR; 2 School of Communication, Hong Kong Baptist University, Kowloon, Hong Kong SAR

**Keywords:** Contact tracing, Structural topic modelling, Surveillance technology, Technological dystopia, Technological pragmatism, Technological utopia

## Abstract

This study investigated people’s ethical concerns of surveillance technology. By adopting the spectrum of technological utopian and dystopian narratives, how people perceive a society constructed through the compulsory use of surveillance technology was explored. This study empirically examined the anonymous online expression of attitudes toward the society-wide, compulsory adoption of a contact tracing app that affected almost every aspect of all people’s everyday lives at a societal level. By applying the structural topic modeling approach to analyze comments on four Hong Kong anonymous discussion forums, topics concerning the technological utopian, dystopian, and pragmatic views on the surveillance app were discovered. The findings showed that people with a technological utopian view on this app believed that the implementation of compulsory app use can facilitate social good and maintain social order. In contrast, individuals who had a technological dystopian view expressed privacy concerns and distrust of this surveillance technology. Techno-pragmatists took a balanced approach and evaluated its implementation practically.

Digital surveillance scholars have argued that the public use of interactive media technologies contributes to turning an information society into an infrastructural surveillance society (Boucher et al., 2018; Lyon, 2022; Marx, 2016). New media technologies can cultivate and empower bureaucratic surveillance to observe, categorize, and control the public in a society (Gandy Jr., 2021). People as consumers in the era of information technology are voluntary to be observed and categorized by consciously providing their personal information through the market’s reward and punishment mechanism (Elmer, 2003). Either bureaucratic or capitalistic surveillance scholarship is based on the study of technology use that is not compulsory. It may be because, although various media technologies have greatly influenced individuals’ everyday lives, to a large extent people can still choose to use the technologies at an individual level. There have been extremely few surveillance technologies that can have wide-range and long-term impacts on almost every aspect of all the citizens’ everyday lives at a societal level.

The recent case of compulsory use of COVID-19 contact tracing apps in some countries/places could provide an opportunity to understand how people perceive a surveillance society constructed through the compulsory use of technology—if the app is not used, individuals are prohibited from accessing almost all service spaces. By referring to Ball (2009), this study intended to contribute to the surveillance paradigm by investigating the subjective experience of surveillance through compulsory app use. To answer calls for more empirical research on surveillance technology (McClain, 2018; Raab, 2002), this study examined the anonymous online expression of attitudes toward the society-wide, compulsory use of a contact tracing app LeaveHomeSafe in Hong Kong as a surveillance technology through the prism of technological utopian/dystopian narratives.

While Bentham’s (1791) panopticon concept dominates the field of surveillance study (Foucault, 1977; Gandy Jr., 2021), this study employed the spectrum of technological utopian and dystopian narratives (involving a balanced technological pragmatic view in the middle ground) instead of the panopticon metaphor as a means to understand the experience of surveillance through the compulsory technology use. The panopticon is a prison where the prisoners are surveilled but do not know when, so that their behavior is controlled and self-disciplined by being watched (Bentham, 1791). The visible and unverifiable power under the panopticon fits the dystopian narrative of a surveillance society (Foucault, 1977) and relates to surveillance through digital technologies (Bossewitch & Sinnreich, 2013; Elmer, 2003). Because of the advancement in interactive media technologies, the concept of surveillance has changed from direct observation and categorization of people to digitized and datafied surveillance (Lyon, 2022). Indeed, *surveillance* as a social practice could be defined as regular and systematic monitoring of personal information for the objectives of management, influence, and protection (Lyon, 2007). Thus, the panopticon metaphor typically applies to the technological dystopian view of surveillance as undesirable social control and privacy invasion, but not the technological utopian aspect of surveillance as care and protection.

The panopticon metaphor could also be associated with protection (such as the maintenance of order and protection of the larger society by avoiding harmful activities in prison) and care (such as the capability of monitoring patients in a hospital setting). Still, the panopticon metaphor tends to focus on the undesirable controlling aspects. However, the spectrum of technological utopian and dystopian narratives can offer balanced optimistic and pessimistic views. Also, the technological utopian/dystopian narratives speculate about the future, which is appropriate for investigating the social influences of new media technologies in our study. In contrast, the panopticon metaphor is mainly rooted in historical systems of surveillance. Finally, the technological utopian/dystopian narratives emphasize human agency, which should fit our investigation of the subjective experience of digital surveillance in this study. The subjective experience could be associated with the perceived technological future.

## The Technological Utopian and Dystopian Spectrum

Social theorists construct the discourses of utopia and dystopia to imagine an ideal or evil society that could offer a standard against which the reality can be compared and criticized (Bauman, 1976; Mannheim, 1966; Marcuse, 1966). The advancement of (surveillance) technology has been ascribed to be one of the possible drivers of creating a utopia (or dystopia) (Braun, 1994; Tegmark, 2017). While understanding how technological utopia/dystopia functions to influence the entire future of human life is theoretically significant (Tegmark, 2017), empirical research on public views toward how a desirable or undesirable society is constructed through the advance of surveillance technology remains scarce.

Utopia in social theory dominantly refers to a perceived ideal society or a better world (Bauman, 1976; Sargent, 1994). The utopia fantasy is idealistic, non-practical, and unrealistic, but could be imagined to be achieved in the near future (Bauman, 1976; Marcuse, 1978). Indeed, utopia functions as a mirror to society and displays flaws by projecting a better alternative (Sargent, 1994). In contrast, dystopia displays undesirable ways of individual life in a society. The creation of this alternative is an identification of social problems that should be avoided, an indictment of the real world, and a demand for improvement (Schulzke, 2014). Both the utopian and dystopian narratives are presented to criticize the status quo, highlight the current social problems, and propose a better world (Mannheim, 1966; Marcuse, 1966). While the majority of literature has followed the utopian/dystopian binary in social theory, this study acknowledged the possibility of the spectrum of technological utopian and dystopian views (including a balanced technological pragmatic view in the middle).

The construction of a better world is most likely actualized by the innovation of technology, but not by the evolution of morality or democratization (Bacon, 1627). Techno-utopians believe that advanced technologies can be utilized to generate social good and maintain social order at one end. In contrast, techno-dystopians have a pessimistic view that people’s freedom will be deprived by technological development at the other end (Dai & Hao, 2018). Indeed, most people are not complete techno-utopians or complete techno-dystopians. In between the two extremes there is a wide range of possibilities. People could have a technological utopian view to a greater extent and a technological dystopian view to a lesser extent, and vice versa. Moreover, some could be neither a techno-utopian nor a techno-dystopian, i.e., people with a technological pragmatic view (Hickman, 2019; Keulartz et al., 2004). Technological pragmatism highlights balanced and realistic practices based on the real-world effectiveness and practical benefits of technologies.

Tegmark (2017) explored a broad range of possible technological utopias and dystopias. One of the main elements among them is the utilization of surveillance technology to construct either an ideal or evil society. An infrastructural surveillance society advanced by mandatory and omnipresent technologies can be perceived as a favorable place because of its all-around protection and care, or an unfavorable place due to the invasions of privacy and freedom. Given that a surveillance society could land on a continuum, the social impacts of surveillance technology are mixed between the two circumstances. In the middle ground, technological pragmatists would practically evaluate the costs and benefits of implementing surveillance technology.

Although scholars’ critics of the status quo by referring to the technological utopia and dystopia discourses are important, it should also be crucial to understand how people (who are the potential techno-utopians, techno-dystopians, and techno-pragmatists) perceive a society constructed through the use of surveillance technology that can actually influence almost every aspect of their everyday lives. As this study investigated the subjective experience of surveillance, the concept of human agency, which emphasizes the degree of control people have in a surveillance society (Hanson, 2020), was taken into consideration. In utopian narratives, human agency is executed through compliance. The concepts of autonomy and freedom may be redefined. The agency is not viewed in relation to freedom or privacy from surveillance but participation and contributions to a safe and ordered society. In dystopian narratives, human agency is significantly diminished. People may feel constantly controlled and manipulated. Within the framework of technological pragmatism, human agency is about making decisions on the use of surveillance technology after evaluating its benefits and drawbacks practically.

The future-orientedness of the spectrum of technological utopian and dystopian narratives could offer insights into the social impacts of surveillance technology. The narratives can extrapolate current technological trends to predict potential future implications (Hanson, 2020). Utopian narratives denote an optimistic vision of the future where surveillance technology will be adopted to benevolently increase societal well-being, whereas dystopian narratives accentuate the damages of sacrificing privacy in the name of safety for future societies. Techno-pragmatists adopt a forward-looking, balanced approach to estimate the long-term benefits and drawbacks of surveillance technology in the future.

The use of COVID-19 contact tracing apps in recent years can represent a case demonstrating how the compulsory adoption of surveillance technology could affect almost all people at a societal level, i.e., people are not allowed to access almost all service spaces without the app. The case could never be a technological utopia/dystopia, but the narratives could be adopted to comprehend how people perceive the current social situation under the pandemic, criticize the status quo, picture a desirable/undesirable alternative, and evaluate its practical results through the compulsory technology use.

## Contact Tracing Apps as a Surveillance Technology

COVID-19 contact tracing mobile apps were created as a technological solution to solve the socio-medical problem during the COVID-19 pandemic (Mann et al., 2022; Vitak & Zimmer, 2020). Contact tracing for COVID-19 refers to the process of recognizing individuals who have contacted infected individuals in person, locating and informing individuals after exposure to a suspected and confirmed case, and maintaining contact with the identified individuals for regular monitoring (World Health Organization, 2022). At a national level, over 49 countries have launched contact tracing apps to track and control the spread of COVID-19 since 2020 (O’Neill et al., 2020). The use of contact tracing apps was voluntary in many countries (e.g., the United States, Germany), and the download rates were low-to-modest (O’Neill et al., 2020). Thus, researchers have developed technology acceptance models to understand factors that predict contact tracing app adoption (Geber & Ho, 2022; Kostka & Habich-Sobiegalla, 2022). In contrast, the adoption of contact tracing apps was compulsory to a certain extent in some countries, such as China, South Korea, and Singapore. Previous studies have examined the public attitudes toward the compulsory use of contact tracing apps in this context (Kim et al., 2021; Liu & Graham, 2021).

The technological utopian and dystopian concepts could be adopted to understand the compulsory use of contact tracing apps as a surveillance technology. Compared with previous studies on contact tracing apps without comprehensively considering the full spectrum of compulsory use, the narratives in this study can be employed to understand views ranging from ideal and practical to unideal perspectives. Previous research found that people perceive that the use of this surveillance technology can generate social good and protect other people (Baik & Jang, 2022), even if they need to sacrifice their privacy to trade off for public health (Liu & Graham, 2021). State surveillance as a form of techno-governance and paternalistic care, as well as collectivistic thought for protecting the majority and facilitating social good, contribute to the technological utopian view. By observing and categorizing non-infected and infected persons into ideal and deviant subjects of the apps, South Korean and Chinese who hold a positive view of compulsory use of contact tracing apps feel cared for through authority control (Kim et al., 2021). An in-depth interview study showed that all (except two) Chinese respondents perceive that contact tracing surveillance in China is necessary for benefiting public health. Chinese people are willing to give up their individual privacy for the collective interest, given that they believe that Chinese cannot possess privacy rights (Liu & Graham, 2021). It is noted that the findings represented attitudes toward the app in the early stages of the pandemic and could not reflect the attitudes during the later phases with long-lasting lockdowns.

On the other hand, the technological dystopian view saw the pervasion of contact tracing apps as a surveillance technology for privacy invasion (Mann et al., 2022; Rowe, 2020). The COVID-19 pandemic could offer opportunities for authorities to amplify their power through new and emergency rules and laws for augmented surveillance technologies (Mann et al., 2022). Privacy concern is one of the main reasons why people refuse to use contact tracing apps (Chan & Saqib, 2021; Kostka & Habich-Sobiegalla, 2022). People do not trust the contact tracing apps if they do not know who have the rights to access and use their personal data gathered by the apps. Contact tracing app users draw an analogy between the surveillance technology and dystopian literature to express their concerns regarding the long-term implications of data misuse (Baik & Jang, 2022). However, people tend to be willing to share data via contact tracing apps if they think that the app use can generate beneficial consequences, including causing fewer infections, offering better health information, and protecting self and others (Habich-Sobiegalla & Kostka, 2022). Thus, the findings may imply that people could have a pragmatic view on the apps.

This study focused on the society-wide, compulsory adoption of a contact tracing app *LeaveHomeSafe* in Hong Kong. The Hong Kong Government launched the LeaveHomeSafe mobile app on 16 November 2020 to offer people in Hong Kong a device for tracing and recording the time of their visits to various places and taxi rides (Office of the Government Chief Information Officer, 2022). The app user will be notified if the person is detected to have visited the same place or taken the same taxi that an infected person has visited or taken on the same day. People needed to upload their COVID-19 vaccination records and testing results to the app. Although the global positioning system is not requested, users have to authorize LeaveHomeSafe to access their cellular network, notification function, and camera lens. The visit history, which is not personally identifiable information, will be stored on users’ smartphones for 31 days before being automatically deleted.

All Hong Kong citizens were required to scan the QR code of the LeaveHomeSafe mobile app before they were permitted to pass into government buildings or offices (including hospitals, libraries, and public markets regulated by the government), restaurants, cinemas, gyms, beauty centers, massage parlors, playgrounds, and theme parks starting in November 2021 (Chau, 2021), and later shopping malls, supermarkets, wet markets and other markets, department stores, hair salons, and religious venues after February 2022 (The Standard, 2022). Accordingly, the use of this contact tracing app covered almost every aspect of all Hong Kong people’s everyday lives at a societal level. Thus, LeaveHomeSafe use could be a close-to-perfect case to comprehend people’s utopian, dystopian, and pragmatic views of a society decorated through the compulsory use of surveillance technology.

The Internet can be a public sphere for people to express their views on social issues (Papacharissi, 2002), such as the compulsory use of contact tracing apps affecting people’s everyday lives enormously. This study explored the expression of attitudes toward the use of LeaveHomeSafe app on Hong Kong anonymous discussion forums. Anonymous discussion forums could be an ecologically valid medium for studying phenomena in natural settings (Ng & Lin, 2022).

This study asked a research question: What are people’s utopian and dystopian views (as well as a pragmatic view) of society-wide, compulsory use of surveillance technology? By referring to Foucault’s (1977) panopticon metaphor and Lyon’s (2022) digitized and datafied surveillance as a social practice, this study was conducted to understand how people with utopian, pragmatic, and dystopian views would differently influence their behavior, subjectivities, and counter-conduct, i.e., the strategies people use to resist ruling power structures and forms of control.

## Method

To capture anonymous online expression regarding LeaveHomeSafe, this study collected online data from four Hong Kong-based anonymous discussion forums, namely LIHKG, Hong Kong Golden Forum (HKGolden), Hong Kong Discuss Forum (DISCUSS), and Baby Kingdom. Users can initiate discussions, provide comments, and ‘like’ others’ posts or comments on the four forums. Almost all users on the four forums were local to Hong Kong, and posts were mainly in written Cantonese. The four forums were chosen because they were the top four most popular anonymous forums in Hong Kong as of September 2022 (Semrush, 2022). Also, the four selected forums covered Hong Kong people with different views. LIHKG and HKGolden users tended to be liberal (Lee et al., 2022; Ng et al., 2022), whereas users on DISCUSS (particularly the current affairs ‘channel’) and Baby Kingdom (a forum mainly for parents) were more likely to be conservative and pro-government (The Encyclopedia of Virtual Communities in Hong Kong, n.d.-b, n.d.-a).

We developed a web crawler to automatically retrieve LeaveHomeSafe-related posts from the four discussion forums. All LeaveHomeSafe-related posts and the subsequent comments thereunder in the four selected forums were collected over 18 months from 11 November 2020 to 10 May 2022 (for details, see Online Appendix A). Table 1 displays the number of LeaveHomeSafe-related comments and merged posts of the four selected discussion forums. Data and syntaxes are available online at https://anonymous.4open.science/r/202301_CompulsoryCTA_codes. Supplementary materials (Online Appendixes) are available online at https://osf.io/dvysk?view_only=b0b460f4385043acb3e960f3a339a84b.

**Table 1:** Number of comments and merged posts containing LeaveHomeSafe of the four selected Hong Kong anonymous discussion forums

Forum	Comments	Merged posts
Mostly negative view toward LeaveHomeSafe		
LIHKG	63,792	1558
HKGolden	21,413	763
Mostly positive view toward LeaveHomeSafe		
DISCUSS	49,319	1061
Baby Kingdom	11,466	312

**Fig. 1:**
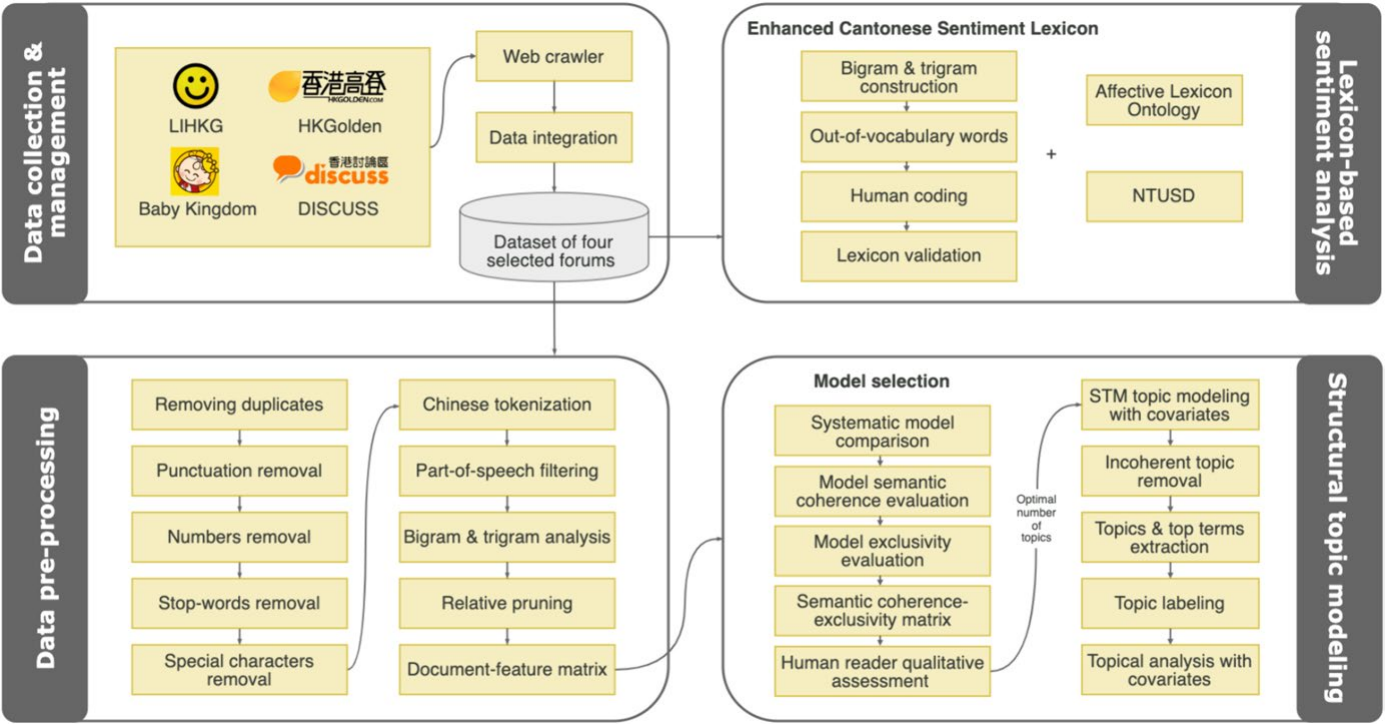
Procedure of Structural Topic Modeling. *Notes.* For details of the procedure, see the Online Appendixes: https://osf.io/dvysk?view_only=b0b460f4385043acb3e960f3a339a84b

We employed structural topic models (Roberts et al., 2014) to explore, determine, and evaluate the patterns and characteristics of topics generated from LeaveHomeSafe-related posts (see Fig. 1). R package *stm* (Roberts et al., 2019) was used to analyze document-level metadata and topic correlations (for more details, see Online Appendix B). This study used *day* (for details, see Online Appendix C), *stance* (forums containing most members expressing positive versus negative views toward LeaveHomeSafe), and *sentiment* (positive versus neutral versus negative sentiments) of each post as metadata (i.e., covariates). By including the three covariates, we estimated and detected how the topical distribution patterns and nuances of the LeaveHomeSafe discussion varied with these covariates of interest.

According to the social identity model of deindividuation effects (Postmes et al., 1998; Spears & Lea, 1994), the anonymous nature of discussion forums could weaken individualistic values, beliefs, and attitudes between in-group members and strengthen in-group members’ collectivistic values, beliefs, and attitudes (Ng et al., 2022). Accordingly, most users in the same anonymous forum should share similar values, attitudes, and views toward a topic, issue, or event. We included forum stance as a covariate because the anonymity of forums could affect the results of forum users’ views toward LeaveHomeSafe. We classified LIHKG and HKGolden as forums containing most members expressing a negative view toward LeaveHomeSafe, and DISCUSS and Baby Kingdom as forums consisting of most members showing a positive view toward LeaveHomeSafe (for the rationales of the classification, see Online Appendix D).

To answer our research question, we analyzed the positive and negative sentiments in forum posts involving LeaveHomeSafe. We developed an Enhanced Cantonese Sentiment Lexicon to identify the sentiments of the posts from the Cantonese-based Hong Kong discussion forums in this study. The reliability and validity of this newly created lexicon were tested. For details, see Online Appendix E.

After importing the LeaveHomeSafe-related dataset into Python 3.8.8, each post underwent the standard pre-processing steps (Maier et al., 2018; Manning et al., 2008) to drop duplicates and remove punctuation marks, numbers, Chinese/Cantonese stop-words, and special characters (e.g., Emojis, web addresses, and HTML-markups). Then, the textual data were separated into word units (i.e., tokens) in the process called tokenization after data cleansing (Manning & Schütze, 1999). For more details, see Online Appendix F.

After that, we implemented relative pruning, removing too ubiquitous or rare tokens, i.e., those appearing in more than 99% or less than 0.3% of the corpus. Those distribution patterns often do not contribute to meaningful topic construction (Grimmer & Stewart, 2013; Maier et al., 2018). After data pre-processing, we obtained 10,539 unique tokens from 3694 posts to construct a document-feature matrix based on the bag-of-words assumption (Turney & Pantel, 2010).

To determine the parameter *K* for the number of topics (Grimmer & Stewart, 2013), we used the function *searchK* from the *stm* package to systematically compare different *K* values (i.e., 5, 10, 15, 20, 25, 30, 35, 40, 45, and 50) by adopting a data-driven iterative approach (Roberts et al., 2019). We examined the quality of individual topic models based on semantic coherence and exclusivity. *K* = 15, 20, 25 and 40 were selected for further qualitative assessment (for details, see Online Appendix G). Based on the combination of quantitative examination (statistical fit measures) and qualitative inspection (human assessments), we selected the final *K* = 25 because the results were better than the other three models. Two topics were discarded for lack of coherence. The remaining 23 valid topics are shown in Table H1, Online Appendix H.

The approach of connecting the topical content and prevalence of structural topic models with covariates is practical for interaction studies that estimate the relationship between covariates and the extracted topics (Roberts et al., 2014, 2016). The purpose of this study was to detect nuanced topical differences from the perspective of the document-level covariates of interest (i.e., stance and sentiment). Therefore, to capture the utopian and dystopian views, we estimated the mean proportion differences between forums mainly expressing positive versus negative views toward LeaveHomeSafe (see Figure H1, Online Appendix H) and positive versus negative sentiments (see Figure H2, Online Appendix H) with a 95% confidence interval. To elucidate the pragmatic view, topics were designated as representing a middle-ground perspective when the topical differences between forums or sentiments did not reach statistical significance (*p* > 0.05, see Figures H1 and H2). The topics should indicate a balanced representation of views toward LeaveHomeSafe without a clear skew toward either positivity or negativity.

## Results

To answer the research question, we analyzed the covariates of interest (i.e., stance and sentiment) and identified topics that reflected technological utopian, dystopian, and pragmatic views toward the society-wide, compulsory use of contact tracing app by referring to the label of each topic and the top 10 prob and FREX terms (see Table H1), as well as the topical contrasts (see Figures H1 and H2). Figures I1, I2, and I3 (Online Appendix I) display topic prevalence for the technological utopian, dystopian, and pragmatic views by month, respectively, with a 95% confidence interval. The combination of the topical contrasts demonstrated a wide range of possibilities and reflected the complexities of the spectrum.

### Technological Utopian View

#### Social Good

Topics 15 and 17 appeared to be concerned with public health and thus generating social good that can benefit the society related to the compulsory implementation of LeaveHomeSafe use. Forum users who tended to use the positive-view forums were more likely to express a positive sentiment of adopting vaccine pass and eHealth to cultivate social good.

The conversations of the two topics mentioning LeaveHomeSafe involved the sharing of people’s vaccination status. Those forum users highlighted ‘LeaveHomeSafe = digital vaccine’ and perceived that a good citizen in a society should use this surveillance technology and should have been vaccinated. The implementation of health code should be accelerated to achieve detailed personal health tracking. In line with a previous study showing that contact-tracking surveillance can reduce the feeling of uncertainty during the pandemic (Baik & Jang, 2022), the forum users felt empowered to deal with the pandemic through the compulsory use of LeaveHomeSafe. The LeaveHomeSafe use could also imply self-surveillance (Humphreys, 2011). People record the time of their visits to different places for replaying later.

Those forum users emphasized that LeaveHomeSafe and COVID-19 vaccines did not only protect themselves, but most importantly their significant others. The surveillance app use can benefit others as well as oneself: ‘I will definitely use this app to protect myself and others. There are too many selfish people in Hong Kong. Their lives are not guaranteed and they still talk about privacy!’ Furthermore, the forum users who had a positive attitude toward LeaveHomeSafe believed that the installation of this surveillance app and registration of eHealth with the vaccine pass can protect the whole society (i.e., Hong Kong) and contribute to the restoration of Hong Kong’s prosperity. For additional quotes, see Online Appendix J.

The findings implied that those forum users perceived the technological surveillance as protection and care (Lyon, 2007, 2022). In consistent with prior research on contact tracking apps demonstrating the form of paternalistic care in pandemic techno-governance (Kim et al., 2021; Liu & Graham, 2021), the LeaveHomeSafe use was perceived as a surveillance technology that can protect self, significant others, and the society.

#### Social Order

Safeguarding social order was another crucial aspect of the technological utopian view on LeaveHomeSafe. We discovered topics related to the adoption of LeaveHomeSafe as a means to maintain social order, including Topics 2, 7, 10, 19, and 24. Pro-LeaveHomeSafe forums dominated the topics concerning the maintenance of social order. While the pro-LeaveHomeSafe forum users showed a positive sentiment toward the government policy and its use for the entry of premises, they expressed a negative sentiment toward the liberals who violated social rules and destroyed social harmony.

Instead of discussing the preservation of social stability directly, people who supported the government (blue/conservatives) denounced yellow/liberals who intended to break the social rules and interrupt the stability of Hong Kong society. Thus, blue/conservatives thought the Hong Kong government needed to use this surveillance technology as a means to safeguard the social order. Yellow/liberals were censured for their rejection of scanning the LeaveHomeSafe app but writing their personal information instead before entering the restaurants. Blue/conservatives gloated over the punishments and arrests of those who did not use this surveillance technology. Specifically, the target of insults was the yellow economic circle (businesses that supported the liberals), particularly the yellow restaurants and people who supported those restaurants. Pro-LeaveHomeSafe individuals believed that the maintenance of social stability and order can be achieved through the compulsory use of this surveillance technology.

Furthermore, Topics 3 and 22 revealed privacy trade-offs to safeguard social order. A forum user said, ‘I don’t mind the health code with tracking function at all,’ considering the trade-off between privacy and social stability. Those forum users had a belief that privacy concerns are trivial because ‘I am just an ordinary citizen, there is nothing to be taken!’ and ‘(those who resist the surveillance app are) selfish and self-interested persons. Those persons abuse their human rights and fear surveillance and disclosure of their whereabouts. They all think they are “national” figures.’ Thus, the contract tracing surveillance was deemed necessary. Even though those forum users understood the potential privacy invasions of LeaveHomeSafe, they thought contact tracing surveillance was necessary for public health (Liu & Graham, 2021). Also, the LeaveHomeSafe use could denote participatory or voluntary panopticon (Humphreys, 2011; Whitaker, 1999): people are voluntary to be surveilled by compulsory technology.

Although the utopia fantasy is idealistic and unrealistic, it functions to picture a better alternative (Mannheim, 1966; Marcuse, 1966). The results implied that people who had a technological utopian view on LeaveHomeSafe thought that the compulsory app use at a societal level can create a better society by generating social good and maintaining social order. The results supported that human agency refers to people’s contributions to a safe and ordered society in the context of technological utopia. Techno-utopians argue that the compulsory use of surveillance technology can benefit the public’s health (Kim et al., 2021; Liu & Graham, 2021). This study extended previous studies by showing that apart from the direct discussion regarding the maintenance of social stability through the compulsory contact tracing apps use, the discourses of techno-governance also include the repression of people having an opposite view.

### Technological Dystopian View

Distrust of surveillance technology and privacy concerns reflected the core of the technological dystopian view on this app. Topics concerning LeaveHomeSafe as an undesirable surveillance technology included Topics 8, 13, 18, and 21. The topics indicated that the users were more likely to use negative-view forums and express a negative sentiment toward the compulsory LeaveHomeSafe use.

In general, those comments showed distrust of this app because of its surveillance function. The LeaveHomeSafe app was perceived as a ‘Trojan Horse. If not, then why are we forced to install this app? It is for the purpose of surveillance’ (see Online Appendix J for additional quotes). Despite their technological dystopian view on this surveillance app, those forum users self-disciplined their behavior to follow the rules and use this app when necessary. The subjective experience of surveillance through the compulsory use of LeaveHomeSafe app fits the panopticon metaphor (Foucault, 1977; Gandy Jr., 2021).

Specifically, those forum users associated the compulsory use of this surveillance technology with some classics of dystopian literature. Some cited George Orwell’s Animal Farm and criticized that Hong Kong people are like animals on a farm. Others modified Aldous Huxley’s Brave New World to ‘Brave New Hong Kong’ to satirize the situation in Hong Kong. Many analogized the compulsory use of this contact tracing app to ‘Big Brother,’ ‘Ministry of Truth,’ and ‘thoughtcrime’ in George Orwell’s 1984. The compulsory LeaveHomeSafe use was perceived by the forum users as the official 1984-mode of mass surveillance. Taking into consideration of privacy concerns, the results of this study were consistent with previous research findings that people make an analogy between surveillance contact tracing apps and the classics of dystopian literature (Baik & Jang, 2022). The difference is the use of contact tracing apps in Baik and Jang’s (2022) study was not mandatory, whereas the use of LeaveHomeSafe as a surveillance technology was compulsory at a societal level.

Similar to the utopia narrative that a technological dystopia is impossible, the imagination of this alternative could be adopted to criticize the current social issues. The findings revealed that people with a technological dystopian view on this surveillance app perceived LeaveHomeSafe as a technology for undesirable social control. The results showed that people felt controlled and the human agency was largely restricted. In line with previous studies criticizing contact tracing apps as a surveillance technology (Mann et al., 2022; Rowe, 2020), techno-dystopians express privacy concerns and distrust of surveillance technology by emphasizing the mass surveillance function of compulsory contact tracing apps.

### Technological Pragmatic View

Our analysis revealed a middle path between techno-utopianism and techno-dystopianism, i.e., technological pragmatism (Topics 1, 9, and 23). Forum users were more likely to express a negative sentiment toward the registration of personal information. Pro-LeaveHomeSafe forum users tended to discuss the elderly’s use and restrictions on restaurants.

Topic 1 encapsulated that users in online forums expressed both frustration and acceptance toward the procedures of entering service spaces like cinemas and restaurants, which include scanning a LeaveHomeSafe code or filling out a form with personal information. One user described the process as cumbersome, especially during peak times, significantly burdening staff and patrons. However, there was an understanding of its necessity, with many eventually opting for the app over manual entry for convenience.

Besides, discussions reflected a blend of criticism and empathy toward the elderly’s challenges in adopting new technology (Topic 9). While some comments were harsh, blaming the elderly for their lack of tech-savviness, others pointed out systemic issues such as the lack of support from community organizations. This discourse underscored a pragmatic understanding of the digital divide and the need for societal adjustments to accommodate all citizens.

Restaurant owners and patrons alike expressed frustration with the changing regulations and impacts on their businesses and daily lives (Topic 23). Nevertheless, these measures were understood as part of a more significant effort to combat the pandemic. A restaurant owner shared, “We are all in this together. While it is a hassle to enforce these rules, it is a small price to pay for everyone’s safety.”

Technological pragmatism (Hickman, 2019; Keulartz et al., 2004) is neither an acceptance of technology’s encroachment on privacy and freedom, nor a blind endorsement of technological solutions. Instead, it recognizes the complexities and trade-offs inherent in the digital age. This discourse challenges the binary of utopian and dystopian narratives, advocating for a more thoughtful, balanced approach to the role of technology in society. The insights from these discussions could offer valuable perspectives on how we might find a balance between technological advancement and the preservation of fundamental human values.

## Discussion

Surveillance technologies (e.g., social credit systems, automated facial recognition systems, and contact-tracing applications) have started to generate society-wide and long-term impacts on almost every aspect of citizens’ everyday lives. The case of LeaveHomeSafe use in this study could provide a chance to comprehend how people perceive an infrastructural surveillance society constructed through the compulsory use of technology. By adopting the spectrum of technological utopian and dystopian narratives, the findings showed that the subjective experience of surveillance technology use could not only be negative because of privacy invasions and unwished mass surveillance, but could also be positive due to its all-around protection and maintenance of social order, as well as be pragmatic evaluating its practical use. Although the case in this study was not a complete technological utopia/dystopia, the narratives were used to understand how people perceive the present social condition under the pandemic and picture an ideal, evil, or pragmatic alternative through the compulsory technology use.

There have been decades-long studies in privacy, surveillance technologies, and social control (Kreissl, 2014), but these studied surveillance technologies were not completely compulsory at a societal level. This study was among the first that could add to the surveillance literature by investigating a compulsory technology causing wide-range and long-term societal impacts. Compared with the literature on non-compulsory technology use, this study provided an opportunity to comprehend how people perceive the compulsory use of surveillance technology. Our results implied that the differences lie in the level of human agency (Hanson, 2020). In the utopian context, while compulsory surveillance presumes a top-down approach that places social demands ahead of individual preferences, non-compulsory surveillance upholds personal choice. In addition, compulsory surveillance in utopias requires a high degree of institutional trust, whereas trust is critical but balanced with measures for individual rights for non-compulsory surveillance. In the dystopian context, compulsory surveillance is represented by apparent, state-mandated control, whereas non-compulsory surveillance entails covert, socially-engineered forms of control determined by consumerism and technology dependence. The pragmatic approach highlights the societal need for compulsory surveillance, but personal preferences and market forces for non-compulsory surveillance.

According to privacy calculus theory (Culnan & Armstrong, 1999), people attribute their value to personal data and evaluate the benefits and risks of sharing data online. If the benefits of disclosing personal information are higher than the risks of privacy disclosure, people are more willing to trade off privacy invasions for transaction benefits. Habich-Sobiegalla and Kostka (2022) found that perceived benefits of sharing data via contact tracing apps (e.g., protecting self and others, reducing infections) are positively correlated with willingness to disclose their personal data (perceived risks and data sharing are not associated). Our findings showed that some surveillance app users were eager to trade off privacy for protection and social stability, reflecting the benefit-centric calculus and trust in governance. However, others perceived that the costs to privacy outweigh the benefits of surveillance, leading to loss of freedoms and abuses of power. The results demonstrated cost-centric calculus and a lack of institutional trust. Future studies could extend or refine the privacy calculus theory by taking into consideration the spectrum of technological utopian and dystopian narratives concerning the compulsory use of surveillance technology. The privacy calculus could strike a balance between the advantages of surveillance and the need to protect personal privacy in the context of technological pragmatism.

The varied reactions to the LeaveHomeSafe app in Hong Kong accentuate the wider implications of introducing surveillance technology in a politically and socially divided society. In a society with substantial fractures, the perception of surveillance technologies could be profoundly polarized. Hong Kong people who trust the government and prioritize public health and safety think that the LeaveHomeSafe app is a necessary tool to manage the spread of COVID-19. For those who are skeptical of the government’s intentions because of the existing political tensions, the app could be interpreted as a tool for enhanced control. Any government-implemented technology could be regarded with distrust in a society with deep-seated political divisions. The fear of using personal data for purposes other than public health could be amplified in the context of recent political unrest.

This study had some limitations. First, the collected anonymous forum text data were subjected to the data cleansing steps (e.g., punctuation, number, emoji, and stop-word removals) to remove noise for subsequent statistical analysis. It should be noted, however, that studying emojis would be a fruitful direction for future work to measure the public’s emotional response. Second, we complemented the misfit of off-the-shelf Chinese sentiment lexicons in Cantonese contexts by creating the Enhanced Cantonese Sentiment Lexicon. Nevertheless, the dictionary approach assumes that texts are mostly context-independent (Grimmer & Stewart, 2013), which may fail to apply to instances where the meaning of words changed by context, the use of irony and negation, and multiple interpretations of the same word. Future research may need to consider an integrated approach to Chinese sentiment analysis by taking the advantages of the bag-of-words model, lexicons, and various natural language processing techniques.

Third, the covariates included in the structural topic models cannot be categorical variables containing more than two values. Thus, we were not able to compare and contrast the four forums. Instead, we dichotomized the forum stance and compared positive-view forums (DISCUSS and Baby Kingdom) with negative-view forums (LIHKG and HKGolden). Future research could use other modeling approaches to solve this issue. Fourth, although some forum users in this study cited dystopian literature, others did not explicitly express their techno-utopian view. Future studies should use other methods to directly ask how people perceive a desirable/undesirable society constructed through surveillance technologies.

## Data Availability

Data and syntaxes are available online at https://anonymous.4open.science/r/202301_CompulsoryCTA_codes. Supplementary materials are available online at https://osf.io/dvysk?view_only=b0b460f4385043acb3e960f3a339a84b.

## References

[CR1] Bacon, F. (1627). *New Atlantis*.

[CR2] Baik J, Jang E (2022). Where horizontal and vertical surveillances meet: Sense-making of US COVID-19 contact-tracing apps during a health crisis. Mobile Media & Communication.

[CR3] Ball K (2009). Exposure: Exploring the subject of surveillance. Information, Communication & Society.

[CR4] Bauman Z (1976). Socialism: The active utopia.

[CR5] Bentham J (1791). Panopticon: Or, the inspection-house. Containing the idea of a new principle of construction applicable to any sort of establishment, in which persons of any Description are to be kept under Inspection.

[CR6] Bossewitch J, Sinnreich A (2013). The end of forgetting: Strategic agency beyond the panopticon. New Media & Society.

[CR7] Boucher P, Nascimento S, Tallacchini M (2018). Emerging ICT for citizens’ veillance: Theoretical and practical insights. Science and Engineering Ethics.

[CR8] Braun E (1994). Can technological innovation lead us to utopia?. Futures.

[CR9] Chan EY, Saqib NU (2021). Privacy concerns can explain unwillingness to download and use contact tracing apps when COVID-19 concerns are high. Computers in Human Behavior.

[CR10] Chau, C. (2021). Covid-19: LeaveHomeSafe gov’t tracing app to be compulsory in all Hong Kong eateries, gyms, cinemas—exemptions unclear. *Hong Kong Free Press*. https://hongkongfp.com/2021/11/24/covid-19-leavehomesafe-govt-tracing-app-to-be-compulsory-in-all-hong-kong-eateries-gyms-cinemas-exemptions-unclear/

[CR11] Culnan MJ, Armstrong PK (1999). Information privacy concerns, procedural fairness, and impersonal trust: An empirical investigation. Organization Science.

[CR12] Dai Y-X, Hao S-T (2018). Transcending the opposition between techno-utopianism and techno-dystopianism. Technology in Society.

[CR13] Elmer G (2003). A diagram of panoptic surveillance. New Media & Society.

[CR14] Foucault M (1977). Discipline and punish: The birth of the prison.

[CR15] Gandy OH (2021). The panoptic sort: A political economy of personal information.

[CR16] Geber S, Ho SS (2022). Examining the cultural dimension of contact-tracing app adoption during the COVID-19 pandemic: A cross-country study in Singapore and Switzerland. Information, Communication & Society..

[CR17] Grimmer J, Stewart BM (2013). Text as data: The promise and pitfalls of automatic content analysis methods for political texts. Political Analysis.

[CR18] Habich-Sobiegalla S, Kostka G (2022). Sharing is caring: Willingness to share personal data through contact tracing apps in China, Germany, and the US. Information, Communication & Society..

[CR19] Hanson CF (2020). Memory and utopian agency in utopian/dystopian literature: Memory of the future.

[CR20] Hickman LA, Fesmire S (2019). Dewey, pragmatism, technology. The oxford handbook of dewey.

[CR21] Humphreys L (2011). Who’s watching whom? A study of interactive technology and surveillance. Journal of Communication.

[CR22] Keulartz J, Schermer M, Korthals M, Swierstra T (2004). Ethics in technological culture: A programmatic proposal for a pragmatist approach. Science, Technology, & Human Values.

[CR23] Kim Y, Chen Y, Liang F (2021). Engineering care in pandemic technogovernance: The politics of care in China and South Korea’s COVID-19 tracking apps. New Media & Society.

[CR24] Kostka G, Habich-Sobiegalla S (2022). In times of crisis: Public perceptions toward COVID-19 contact tracing apps in China, Germany, and the United States. New Media & Society.

[CR25] Kreissl R (2014). Assessing security technology’s impact: Old tools for new problems. Science and Engineering Ethics.

[CR26] Lee FLF, Liang H, Cheng EW, Tang GKY, Yuen S (2022). Affordances, movement dynamics, and a centralized digital communication platform in a networked movement. Information, Communication & Society.

[CR27] Liu C, Graham R (2021). Making sense of algorithms: Relational perception of contact tracing and risk assessment during COVID-19. Big Data & Society.

[CR28] Lyon D (2007). Surveillance studies: An overview.

[CR29] Lyon D (2022). Surveillance. Internet Policy Review.

[CR30] Maier D, Waldherr A, Miltner P, Wiedemann G, Niekler A, Keinert A, Pfetsch B, Heyer G, Reber U, Häussler T, Schmid-Petri H, Adam S (2018). Applying LDA topic modeling in communication research: Toward a valid and reliable methodology. Communication Methods and Measures.

[CR31] Mann M, Mitchell P, Foth M (2022). Between surveillance and technological solutionism: A critique of privacy-preserving apps for COVID-19 contact-tracing. New Media & Society.

[CR32] Mannheim K (1966). Ideology and utopia.

[CR33] Manning CD, Raghavan P, Schütze H (2008). Introduction to information retrieval.

[CR34] Manning CD, Schütze H (1999). Foundations of statistical natural language processing.

[CR35] Marcuse H (1966). Eros and civilization: A philosophical inquiry into Freud.

[CR36] Marcuse H (1978). The aesthetic dimension.

[CR37] Marx GT (2016). Windows into the soul: Surveillance and society in an age of high technology.

[CR38] McClain N (2018). The horizons of technological control: Automated surveillance in the New York subway. Information, Communication & Society.

[CR39] Ng Y-L, Lin Z (2022). Exploring conversation topics in conversational artificial intelligence–based social mediated communities of practice. Computers in Human Behavior.

[CR40] Ng Y-L, Song Y, Huang Y (2022). Supportive and uncivil expressions in discussions on out-groups by in-group members in anonymous computer-mediated communication. Telematics and Informatics.

[CR41] O’Neill, P. H., Ryan-Mosley, T., & Johnson, B. (2020). A flood of coronavirus apps are tracking us. Now it’s time to keep track of them*.**MIT Technology Review*. https://www.technologyreview.com/2020/05/07/1000961/launching-mittr-covid-tracing-tracker/

[CR42] Office of the Government Chief Information Officer. (2022). *LeaveHomeSafe*. https://www.leavehomesafe.gov.hk/en/

[CR43] Papacharissi Z (2002). The virtual sphere: The internet as a public sphere. New Media & Society.

[CR44] Postmes T, Spears R, Lea M (1998). Breaching or building social barriers? SIDE effects of computer-mediated communication. Communication Research.

[CR45] Raab CD (2002). Surveillance: The need for research evidence. Information, Communication & Society.

[CR46] Roberts ME, Stewart BM, Airoldi EM (2016). A model of text for experimentation in the social sciences. Journal of the American Statistical Association.

[CR47] Roberts ME, Stewart BM, Tingley D (2019). stm: An R package for structural topic models. Journal of Statistical Software.

[CR48] Roberts ME, Stewart BM, Tingley D, Lucas C, Leder-Luis J, Gadarian SK, Albertson B, Rand DG (2014). Structural topic models for open-ended survey responses. American Journal of Political Science.

[CR49] Rowe F (2020). Contact tracing apps and values dilemmas: A privacy paradox in a neo-liberal world. International Journal of Information Management.

[CR50] Sargent LT (1994). The three faces of utopianism revisited. Utopian Studies.

[CR51] Schulzke M (2014). The critical power of virtual dystopias. Games and Culture.

[CR52] Semrush. (2022). *Top websites*. https://www.semrush.com/website/top/hong-kong/all/

[CR53] Spears R, Lea M (1994). Panacea or panopticon? The hidden power in computer-mediated communication. Communication Research.

[CR54] Tegmark M (2017). Life 3.0: Being human in the age of artificial intelligence.

[CR55] The Encyclopedia of Virtual Communities in Hong Kong. (n.d.-a). *Baby Kingdom*. https://evchk.fandom.com/zh/wiki/親子王國討論區

[CR56] The Encyclopedia of Virtual Communities in Hong Kong. (n.d.-b). *Hong Kong Discuss Forum, current affairs channel*. https://evchk.fandom.com/zh/wiki/香港討論區時事新聞區

[CR57] The Standard. (2022). *Hong Kong will ban the unvaccinated from malls, supermarkets, wet markets: Carrie Lam*. https://www.thestandard.com.hk/breaking-news/section/4/186885/

[CR58] Turney PD, Pantel P (2010). From frequency to meaning: Vector space models of semantics. Journal of Artificial Intelligence Research.

[CR59] Vitak J, Zimmer M (2020). More than just privacy: Using contextual integrity to evaluate the long-term risks from COVID-19 surveillance technologies. Social Media + Society.

[CR60] Whitaker R (1999). The end of privacy: How total surveillance is becoming a reality.

[CR61] World Health Organization. (2022). *Contact tracing and quarantine in the context of COVID-19: Interim guidance*. https://www.who.int/publications/i/item/WHO-2019-nCoV-Contact_tracing_and_quarantine-2022.1 37184162

